# The Effect of Shadow Education on Hong Kong Student Wellbeing: Evidence From PISA 2018

**DOI:** 10.3389/fpsyg.2022.860179

**Published:** 2022-05-10

**Authors:** Han Liang, Zhongxing Wang, Wensheng Wu

**Affiliations:** ^1^Jing Hengyi School of Education, Hangzhou Normal University, Hangzhou, China; ^2^College of Education, Qufu Normal University, Qufu, China

**Keywords:** shadow education, wellbeing, hierarchical linear modeling, PISA 2018, Hong Kong

## Abstract

This study investigates the effect of shadow education on Hong Kong student wellbeing. The data were extracted from PISA 2018 (Programme For International Student Assessment 2018) of Hong Kong, and HLM analysis was conducted with student and school dimensions as the independent variables and student wellbeing as the dependent variable. The results in the student dimension showed that students attending shadow education had a significantly higher level of wellbeing than students who did not attend, and in the school dimension, that school competition climate had a significant impact on students' wellbeing; however, shadow education caused by schoolwork pressure and shadow education support appeared to have no significant impact on wellbeing. Furthermore, there was an interactive effect between competition climate and shadow education time which negatively affected wellbeing.

## Introduction

Shadow education has become a predominant phenomenon, first appearing in Asian countries (e.g., Dawson, 2010; Bray et al., 2015; Ozaki, 2015; Kim, 2016; Kim et al., 2018; Zhang and Bray, 2020) and thenceforth spreading globally (Bray and Kwo, 2014; Paramita, 2015). In the process, certain research issues have been highlighted, such as curriculum (Kim and Jung, 2019), policy making (Lee et al., 2010), and determinants (Takashiro, 2021) as the key themes. The implications of shadow education vary: on the positive side, it enhances learning efficacy and provides a constructive environment in society (Manzon and Areepattamannil, 2014); on the negative side, it reduces playtime for children (Choi and Park, 2016) and broadens the inequity gap, causing financial burden (Bray, 1999; Jokić et al., 2013). Referring to the extant literature and using PISA 2018 data, this study explores the construction of linear regression equation models with full consideration for the endogenous factor type, thereby revealing the specific effect of shadow education on student wellbeing. This study provides a certain quantitative basis for countries and regions other than Hong Kong in their formulation of sound policy and their methods of selection with respect to the essential reasons for students attending shadow education and also the association between shadow education and student wellbeing.

### Shadow Education

Stevenson and Baker (1992) used the term *shadow* to denote the strong connection between educational allocation rules and non-formal schooling, implying that such rules tend to be hidden in countries such as Taiwan (Lin, 1983) and Hon Kong (Sweeting, 1983). In fact, in some educational systems (e.g., in Japan and China), these activities make up a large, open enterprise. Private supplementary curriculum tutoring beyond the hours of formal schooling is regarded as shadow education (Bray, 1999, 2009; Buchmann et al., 2010). Shadow education, which is also called private tutoring and additional instruction interchangeably, occurs because of fierce competition for entry into college, education inequity, and the high college aspirations of students. Kim and Jung (2019) disclosed five shadow education practices in South Korea as follows: home-visit private tutoring, private tutoring in institutes, subscribed learning programs, internet-based private tutoring, and after-school programs. Zhang and Bray (2020) denoted three modes of shadow education, consisting of providers, forms, and seasons. For example, providers include individuals and institutions; forms include online, dual-tutor, and offline.

The direct purpose of shadow education is to improve academic performance but studies have shown that its impact on performance is not consistent. Kim (2015) found that private tutoring expenditure appeared to have significant effects on standardized test achievement but not have statistically significant effects on school performance achievement. Guill and Bos (2014) showed that neither hierarchical regression analysis nor propensity scores matching approach revealed any positive effect of private tutoring on student achievement when several cognitive, motivational, family, and school context covariates were controlled for.

PISA investigated extracurricular activities and additional instruction in 2018, which is part of shadow education, in each educational cycle. Useful results were generated. Extracurricular activities mainly cover activities in-school, whereas additional instruction refers to extra time outside of mainstream school hours; such time may be provided at private institutions, at home, or elsewhere. PISA research has been developed for more than 20 years with subjects and time as two essential variables used to evaluate shadow education. Examples of questions posed are as follows: in the year 2000, “During the last 3 years, have you attended any of these special courses outside of your school to improve your results?” and, in 2003, “On average, how many hours do you spend each week on the following?” In 2015, the term “additional instruction” and question items such as “In this school year, approximately how many hours per week do you attend additional instruction in the following domains in addition to mandatory school lessons?” were included in the framework. Questions and instructions that compare in-school and outside-school factors were included in PISA 2015 such as “Compare your lessons at school and your additional science instruction?” and “Where are the following teacher characteristics more likely to occur?” In PISA 2018, motivation information was sought through items such as “Why do you attend additional instruction in this school year.”

### Student Wellbeing

Wellbeing is a complex, multidimensional construct that cannot be properly measured by a sole indicator in a single domain (Borgonovi and Judit, 2016). The concept of wellbeing concerns optimal psychological functioning and experience (Ryan and Deci, 2001). Researchers, although finding that the formulation of a universal concept of wellbeing was challenging, attempted nevertheless to build up various conceptual models. For example, Scanlon (1998) based his contractualism on a broader account of wellbeing; Sumner (1998) pointed out that virtue is the most important constituent of wellbeing. Student wellbeing, defined as students' overall development and quality of life, is increasingly accounted for in education policy (OECD, 2017). Especially for young pupils in all-level schools, educational outcomes have a strong relation with wellbeing. Konu and Rimpelä (2002) attempted to construct a school wellbeing model by identifying four indicators to measure student wellbeing, that is, school conditions (having), social relationships (loving), means for self-fulfillment (being), and health status. Ryan and Deci (2001) proposed that hedonic wellbeing depicts spontaneous feelings of happiness and that eudaimonic wellbeing concerns deeper self-realization. The varying views of wellbeing have shaped two distinct, yet overlapping, perspectives for empirical inquiry, namely, subjective and psychological wellbeing (Ryan and Deci, 2001). However, the above definitions of student wellbeing involve spontaneous feelings, self-realization, etc. Thus, it is clear that student wellbeing is a complex, multidimensional construct that cannot be properly measured by a sole indicator in a single domain (Borgonovi and Pál, 2016).

OECD (2017) proposed the *how's life* framework to measure wellbeing using 11 dimensions under two broad headings. The “Material Condition” covers a dimension grounded in market transactions: income and wealth, jobs and earnings, and housing. “Quality of Life” encompasses factors that are essential to welfare: health status, work-life balance, education and skills, social connections, civic engagement and governance, environmental quality, personal security, and subjective wellbeing. The *Framework for the Analysis of Student Well-Being* (FASWB) proposed by PISA is one of their widely utilized tools, which measure student wellbeing. In 2015, PISA became the first large-scale testing program to extract student wellbeing by consisting of independent indicators, and it developed the FASWB in 2018. The questionnaire covers the dimensions of life as a whole, self-related wellbeing (health, education and skills, and psychological functioning), school-related wellbeing (social connections and schoolwork), and wellbeing outside of school (social connections, material conditions, and leisure time), all of them also representing objective and subjective perceptions, affect, and satisfaction perspectives. OECD (2019) categorized student wellbeing into five domains, namely, cognitive, psychological, physical, social, and material wellbeing. The nomination of student wellbeing as a key research field was due to various factors such as lengthy schooling, high suicide rate (prompted by fierce competition), dislike of schooling, diminished school engagement, and school anxiety (Natsuaki et al., 2009; McGill et al., 2012). In 2021, PISA extended its survey to assess teachers' occupational wellbeing, thus building up a student-teacher binary research base for further studies.

### Research Questions

As mentioned earlier, shadow education consists of various factors, such as in-school vs. out-school, public vs. private, online vs. offline, instruction by teacher vs. by tutor, and during semester vs. during vacation. This study adopts a specific conceptional definition from PISA 2018, *additional course*, which is presented in our student questionnaire and which refers to time spent on extra courses as well as which kinds of subject students attend after school. Student wellbeing in our study includes affective, life meaning, and belonging to school. We set out to explore the factors influencing student wellbeing, under the framework of PISA 2018, in order to shed light on how shadow education influence student wellbeing and to provide an empirical basis for further research. In terms of the factors affecting student wellbeing, “student” and “school” would be extracted as dimensions and would form the key issues in our research. The following questions underpinned our study:

(1) What is the current level of shadow education participation and wellbeing for students in Hong Kong?
(2) What is the relationship between student participation in shadow education and wellbeing? Specifically, is the effect positive or negative?
(3) What causes the current mediating effect of shadow education on student wellbeing?

## Methods

### Participants

A total of 6,037 students completed the PISA 2018 survey from 152 Hong Kong schools (7.2% from government; 78.1% from aided or capital; 2.3% from private or international; 12.4% from a direct subsidy scheme). Students' age ranged from 15.25 to 16.25 years, with an average of 15.73 years. The sample contained 2,955 (48.9%) female respondents and 3,082 (51.1%) male respondents.

### Variables

The variables utilized were taken from the PISA 2018 questionnaire and consisted of student, school, education career, and student wellbeing domains.

#### Dependent Variables

Student wellbeing was targeted as being one dimension of the study's dependent variables and consisted of affective wellbeing, meaning in life, and sense of belonging to school from PISA 2018 data.

(1) Affective wellbeing refers to positive and negative effects and is a dimension of overall student wellbeing. This study focuses on positive affect, including items such as “Thinking about yourself and how you normally feel: How often do you feel as described below? Happy, Lively, Proud, Joyful, Cheerful.”
(2) The scale for meaning in life contains three question items: “My life has clear meaning or purpose,” “I have discovered a satisfactory meaning in life,” and “I have a clear sense of what gives meaning to my life.”
(3) Sense of belonging to school encompasses six question items such as “I feel like an outsider (or left out of things) at school,” “I make friends easily at school,” “I feel awkward and out of place in my school,” “Other students seem to like me,” “I feel lonely at school” and “I feel like I belong at school.”

#### Student Variables

Scores on demographic variables such as gender and ESCS (index of economic, social, and cultural status) were obtained from respondents. The shadow education variables used in this study encompassed five questions concerning items, which are regarded as nominal variables in regression analysis. They consist of items such as “Do you currently attend additional instruction?” For numeric variables in regression analysis, there was only one question item, i.e., “On the most recent day you attended school, how long did you study after leaving school?”

#### School Variables

Items on the type of school (public or private) were responded to by school principals. Items that reflected positive attitudes toward shadow education comprised those such as “Why do you attend additional instruction in < test language> this school year? My teachers recommend it,” this latter item represents an incentive for attendance at shadow education. With regard to competition in school, four items were used, such as “Students seem to value competition,” “Students seem to share the feeling that competing with each other is important,” “Students feel that they are being compared with others” and “It seems that students are competing with each other.” Schoolwork pressure was represented by one item: “Why do you attend additional instruction in < test language> this school year? I want to learn more.”

### Modeling

This study performs data processing utilizing SPSS.25 preliminary sorting and transformation and employs HLM6.08 data analysis on the significant factors of wellbeing in Hong Kong for two horizontal linear model analyses. To find out the effect of shadow education on student wellbeing, two-level HLM is used to examine the relationships between student and school-level variables.

#### Zero Model

First, the zero model was established to separate the components of student wellbeing (i.e., affective wellbeing, life meaning, and belonging to school) into the component caused by individual differences and the component caused by inter-group divergence: thus, without adding independent variables, this study explores whether there were significant inter-school differences in shadow education in Hong Kong.

- Level-1 equation:Y_ij_ = β_0j_+r_ij_, r_ij_~N(0,δ^2^)
- Level-2 equation:β_0j_ = γ_00_+μ_0j_, μ_0j_~N(0,τ_∞_)

*Y*_*ij*_ represents the wellbeing of student *i* in school *j*, β_0*j*_ represents the mean score of school *j*; *r*_*ij*_ represents the random effect of student *i* in school *j*, μ_0*j*_ represents the random effect of school *j*, δ^2^ represents student in-school divergence on student wellbeing, and τ_∞_ represents student between-school divergence on wellbeing.

#### Random Effects Covariance Model

By adding student-level variables into the Level-1 equation, including student background variables (i.e., gender and ESCS), shadow education attendance, and shadow education time, and assuming that the effect of student variables is constant between schools, the effect of student characteristics on student wellbeing can be observed by separating within the school and between school.

- Level-1 equation: Y_ij_ = β_0j_ + β_1j_ Gender + β_2j_ ESCS + β_3j_ shadow education attendance + β_4j_ shadow education time + r_ij_.

where β_0_ is the Level-1 equation's range, representing the average level. β_1_… β_4_ are partial regression coefficients at the student dimension and represent the effects on student wellbeing.

#### Non-random Intercept Model

Based on the random effects covariance model, by adding school-level variables into the β_0_ equation, including school type, schools supporting shadow education, and shadow education caused by academic pressure, we can analyze the differences in student wellbeing among different schools.

β_0j_ = γ_00+_γ_01_ School type + γ_02_ Supportive of shadow education + γ_03_ Competition climate + γ_04_ Schoolwork pressure + μ_0j_…

#### Integrated Model

School-dimension variables are added to the slope β_1_…β_4_ of the Level-1 equation to construct a two-level complete analysis model which includes the interaction component.

Student dimension: Y_ij_ = β_0j_ + β_1j_ Gender + β_2j_ ESCS + β_3j_ Attendance of shadow education + β_4j_ SE time + r_ij_…

School dimension: β_0j_ = γ_00+_γ_02_ School type + γ_03_ Supportive of shadow education + γ_03_ Competition climate + γ_04_ Schoolwork pressure +μ_0j_

β_4j_ = γ_40+_γ_41_ School type + γ_42_ Supportive of shadow education + γ_43_ Competition climate + γ_44_ Schoolwork pressure + μ_0j_

where γ_00_-γ_05_…… γ_40_-γ_44_ are secondary hierarchy regression coefficients that represent the effects of shadow education on a school level and variables including supportive of shadow education, competition climate, and schoolwork pressure.

## Results

### Descriptive Results

#### Description of Different Dimensions

Table 1 shows that students in Hong Kong generally have negative feedback regarding their affective wellbeing (−0.06), life meaning (−0.03), and belonging to school (−0.39). Student wellbeing is generally low, at least in these three dimensions. On average, Hong Kong students study for approximately 4 h after school every day. Table 1 shows that teachers on average recommend that students attend remedial courses (mean = 0.27) and students' attendance at shadow education may derive from self-pressure (mean = 0.65) rather than school competition (mean = 0.13).

**Table 1:** Descriptive analysis of different dimensions.

**Dimension**	**Predicator**	**Mean**	**Std. deviation**	**Maximum**	**Minimum**
	Positive affect	−0.06	0.94	1.24	−3.01
Dependent	Life meaning	−0.03	0.94	1.74	−2.15
Variables	Belonging to school	−0.39	0.70	2.72	−3.24
	ESCS	−0.52	1.02	3.37	−6.52
Student	SE attendance	1.8	0.40	2	1
Dimension	SE time	4.15	3.10	11	1
	Supportive of SE	0.27	0.44	1	0
School dimension	Competition climate	0.13	0.83	2.01	−2.35
	Schoolwork pressure	0.65	0.48	1	1

*SE, shadow education*.

#### Description of Subjects' Distribution of Shadow Education

The frequency analysis (refer to Table 2) shows no significant differences among the choices of different subjects for the Hong Kong students. Overall, enrichment courses are preferred over remedial courses. Among enrichment courses, the mathematics option has the highest participation rate, and the science option has the lowest; among remedial courses, mathematics has the highest participation rate, and test language has the lowest. These results may be explained by the fact that for enrichment courses, mathematics is considered the most difficult course whereas science is comparatively easy to understand. The overall attendance at additional courses in Hong Kong is around one-quarter attendance, which demonstrates that “seeking academic excellence” is the key driver in attendance at shadow education. Based on the results described above, the Hong Kong students who attend additional courses may be described as follows: they comprise a small proportion of students who were left behind in academic achievement and who wish to learn mainly about mathematics in order to enrich their knowledge and enhance their testing skills, thereby obtaining a high ranking in their school.

**Table 2:** Description of different subjects.

**Subjects SE**		**Frequency**	**Percent**
Test language	Enrichment	1,638	27.1%
	Remedial	1,070	17.7%
Mathematics	Enrichment	2,174	36%
	Remedial	1,522	25.2
Science	Enrichment	1,335	22.1%
	Remedial	1,074	17.8%
Foreign language	Enrichment	1,808	29.9%
	Remedial	1,272	21.1%
Study skills		1,751	29%

### Student Wellbeing Differences in and Between Schools

The cross-level coefficient ρ = τ∞/(τ∞+δ2) = 147.083/(147.083 + 163.547) = 0.473 shows that 47.3% of the total variance of the dependent variable affective student wellbeing derives from differences between schools, whereas 52.7% is due to differences within schools. When the dependent variable is belonging to school, the cross-level correlation coefficient is ρ = τ∞/(τ∞ + δ2) = 0.585, indicating that 58.5% of the total variance comes from differences between schools, and 41.5% comes from differences within schools. When the dependent variable is life meaning, the cross-level correlation coefficient is ρ = τ∞/(τ∞ + δ2) = 87.367/(105.347 + 87.367) = 0.453, indicating that 45.3% of the total variance comes from differences between schools and 54.7% is derived from differences within the school. According to Cohen's (1988) definition, ρ > 0.138 indicates a high degree of correlation. Therefore, the differences between schools cannot be ignored; it suggests that student wellbeing can be explained partly by factors within the school and partly by factors between schools.

### Effects on Student Wellbeing From the Student Dimension

In our analysis of student gender, ESCS, shadow education attendance, and outside-school learning time, the random effects covariance model analysis (refer to Table 3) shows that there were no significant differences for the dependent variable effect. For the variables, gender (*p* = 0.255 > 0.05), ESCS (*p* = 0.276 > 0.05), and shadow education attending time (*p* = 0.442 > 0.05), there were also no significant differences but for shadow education attending (*p* = 0.002 < 0.05), there was a significant positive predictive effect for affective wellbeing. This result indicates that: (1) there are no significant differences in affective wellbeing for gender and ESCS. The correlation between shadow education attendance and affective wellbeing reached significance. The dependent variable belonging to school did not have a significant effect on gender (*p* = 0.294 > 0.05), ESCS (*p* = 0.539 > 0.05), or shadow education time (*p* = 0.147 > 0.05). Results for the variable shadow education attendance were significant (*p* = 0.035 < 0.05). (2) There were no significant differences in belonging to a school for gender and family economic and cultural status; there was no correlation between outside-school learning time and belonging to school. There were no significant differences for life meaning in ESCS (*p* = 0.065 > 0.05) and shadow education time (*p* = 0.198 > 0.05), but there were significant differences for gender (*p* = 0.031 < 0.05) and shadow education attendance (*p* = 0.000 < 0.01). (3) There were no significant differences in life meaning for students with different family economic and cultural statuses; shadow education time was negatively correlated with effective wellbeing, although this finding was not significant; students attending shadow education have a higher level of psychological resilience than those who do not attend.

**Table 3:** Estimated variations in-and-between schools.

**Random Effects**	**Variations between school**		**Variations in school**	
	**SD**	**VC**	**SD**	**VC**
Positive Affect	12.13	147.08	12.79	163.55
Belonging to school	12.38	153.16	10.41	108.33
Meaning in life	12.55	157.45	10.81	116.78

*SD, standard deviation; VC, variance component*.

### Effects on Student Wellbeing of School Dimension

When school dimension, school type, supportive of shadow education, schoolwork pressure, and competition climate were gradually added and a non-random intercept model analysis was performed (refer to Table 3), the results showed that when the dependent variable was affective wellbeing, school type (*p* = 0.579 > 0.01), supportive of shadow education (*p* = 0.953 > 0.01), and schoolwork pressure to attendance of shadow education (*p* = 0.184 > 0.01) were significant. The competition climate (*p* = 0.012 < 0.05) variable had a significant impact on affective wellbeing, suggesting that competition climate in the school is conducive to affective wellbeing. When the dependent variable is belonging to school, the school type (*p* = 0.502 > 0.01), supportive of shadow education (*p* = 0.938 > 0.01), and schoolwork pressure leading to attendance of shadow education (*p* = 0.097 > 0.01) had no effect on belonging to school. Competition climate (*p* = 0.009 < 0.01) had a significant impact on belonging to school. When the dependent variable is life meaning, the types of school (*p* = 0.496 > 0.01), supportive of shadow education (*p* = 0.734 > 0.01), and schoolwork pressure leading to attendance of shadow education (*p* = 0.077 > 0.01) had no significant impact on life meaning. In terms of the variable of competition climate (*p* = 0.011 < 0.05), the competition climate has a significant impact on life meaning, indicating that the competition climate can stimulate students' life meaning.

### Interactive Effects of School and Student Dimensions on Student Wellbeing

The student is a key component in school, so the characteristics of shadow education in the school dimension can affect student wellbeing to a certain extent.

In the cross-level interaction of the integrated model (refer to Table 4), when the dependent variable was wellbeing, school supportive of shadow education was shown to play a moderating role in attendance of shadow education in affective wellbeing. In that case, supportive of shadow education had a negatively weakening effect on attendance of shadow education and affective student wellbeing. For each additional unit of supportive shadow education, the impact of attendance shadow education on affective student wellbeing decreased by one. These findings suggest that if a school strongly supports students' shadow education, their affective wellbeing will increase.

**Table 4:** Effects of shadow education on student wellbeing with HLM regression analysis.

**Predicator**	**Positive Affect**		**Belonging to school**		**Life meaning**	
	***P***	**SE**	***P***	**SE**	***P***	**SE**
Fixed-effect intercept	1.59	1.31	0.22	1.27	0.24	1.12
**Student dimension**						
Gender	0.43	0.38	0.62	0.30	0.64^*^	0.30
ESCS	0.26	0.24	0.13	0.20	0.28	0.15
Attendance of SE	0.50^**^	0.18	0.62^*^	0.15	0.64^**^	0.15
SE time	−0.02	0.02	−0.03	0.02	−0.07	0.05
**School dimension**						
School type	−3.94	5.04	−4.10	6.10	−6.01	6.39
Supportive of SE	−7.09	6.84	−0.40	5.13	−2.17	4.77
Schoolwork pressure	44.55	18.12	−7.02	17.93	−4.03	7.71
Competition climate	30.96^*^	10.63	30.46^**^	11.46	29.90^*^	12.45	

* *p < 0.05;*

** *p < 0.01*.

When the dependent variable was belonging to school, attendance of shadow education caused by competition climate was shown to play a moderating role between the length of attendance at shadow education and belonging to school. The relationship has a negative weakening effect. In schools with high attendance at shadow education caused by competition, students with longer extracurricular hours have a stronger sense of belonging to school.

When the dependent variable is life meaning, attendance at shadow education caused by competition has a negative effect on the relationship between the length of extracurricular study and life meaning. It is concluded that in schools with high attendance at shadow education caused by the competition climate, students with longer extracurricular hours have a strong sense of life meaning; and in schools with high attendance at shadow education caused by support for shadow education, students with longer extracurricular hours have a stronger sense of life meaning.

## Conclusion and Discussion

### Effects on Student Dimension

The results suggest that attendance at shadow education has a significant impact on affective student wellbeing, belonging to school, and life meaning. The length of extracurricular study has a negative association with a sense of affective student wellbeing, belonging to school, and life meaning. Students who attend shadow education have a higher level of affective wellbeing, belonging to school, and meaning in life than students who do not. Female students have a higher level of life meaning than male students. The result testifies that shadow education is not detrimental; rather, attending shadow education improves student wellbeing in Hong Kong. Influenced by a mixed model of Confucian and western cultures, Hong Kong students demonstrate some features of Confucian culture. Participants from the Confucian cultural sphere have a highly positive parenting style, affecting the relationship between education and social achievement. The positive impact of shadow education on students' wellbeing has been greatly influenced by this educational boom. As mentioned earlier, the incentive for attendance at shadow education derives mainly from the students themselves, which means that the students who attend shadow education are mostly self-motivated. Kevin (2020), who conducted narrative research on learning under shadow education in Hong Kong, pointed out that shadow education plays a pivotal role in the advancement and future careers, helping students in Hong Kong successfully complete their education. Kevin (2020) also found that most students accept shadow education, which may be viewed as a necessity, and that those students may not feel disadvantaged. The results of this study are also consistent with Cayubit et al. (2014)'s findings, that is, that the inferiority and inadequacy related to learning will be transformed into a sense of efficacy, self-confidence, and high self-esteem after attendance at shadow education. Thus, the provision of private education has a rational basis and is an inevitable trend, consistent with the results of studies in Western countries (Bray, 2009; Burch, 2009; Davies and Guppy, 2010; Silova, 2010). Zhang and Bray (2020) highlighted how learning and teaching are personalized processes in which the private sector can provide practical answers to the problems in public schools. As a result, attendance at shadow education will indirectly improve happiness, with the impact not limited to those often reported in the literature.

### Effects From the School Dimension

Among the variables of school shadow education, our results suggested that school competition climate has a significant impact on affective student wellbeing, belonging to school, and life meaning, whereas the three variables of school type of shadow education caused by schoolwork pressure and shadow education support have no significant impact on affective student wellbeing, school sense of belonging, and life meaning. Bray (2013) also found that competition climate has a significant impact on student wellbeing, and students in schools with a strong competition climate are more likely to fear failure, so they tend to relieve their emotional pressure by attending shadow education.

We found that shadow education attendance due to schoolwork pressure and school support did not have a significant impact on students' wellbeing; Bray (2013), however, found that students who attend shadow education due to schoolwork pressure can significantly improve their academic performance. Another key finding in this study was that most of the Hong Kong students who attend shadow education do so because of their own preferences instead of recommendations from teachers. This finding is in accordance with Zhan et al.'s (2013) research which showed that Hong Kong students believe that shadow education helps them cope with examinations more efficiently compared with mainstream education in schools. It can be inferred that shadow education prompted by school factors has a significant effect on testing scores but not on student wellbeing: students who are in a fierce competition climate and who put more time into shadow education will have lower levels of wellbeing.

## Implications

The key result of this study shows that shadow education positively affects student wellbeing, as opposed to previous studies concerning its negative effects on student psychological health and affect. Education expectations may play a mediating role in the relationship between shadow education and student wellbeing. Because Hong Kong's society is influenced by Confucian culture, most students expect to study hard in order to positively influence their fate. Some researchers have found that fanaticism toward their education is the most striking feature of Asian students (Weiping and Kouyan, 2020). High participation in shadow education is one of the manifestations of such fanaticism about education. Shadow education connecting in- and out-schooling is highly regarded by “fanatical” or highly motivated university students because it helps students fulfill their learning objectives. Therefore, in high competition climates, certain shadow education practices can enhance student wellbeing despite the resulting experience of combined enjoyment and suffering: having a purpose for suffering can transform its nature. Motivation for shadow education can be divided into two levels. The first level concerns goals, that is, achievement of better test results: students perform consistently on this level. The second level concerns self-driven motivation vs. school-driven or parent-driven motivation. Shadow education that is self-driven fosters student wellbeing, whereas education driven by parents or schools is detrimental to student wellbeing.

If shadow education starts from the basic point of maintaining social equity and guaranteeing the right to education of all students, it can foster student wellbeing in more domains and achieve the all-round development of students. Some countries and organizations decide the future direction of education through policy adjustment. In this process, student wellbeing has always been a key issue for consideration. As stated earlier, if education is self-driven, the government will support the students involved. In contrast, governments that are mainly driven by parents or external forces to get children into exam-oriented education are generally not supportive of students (China, Ministry of Education, 2018; Pakistan, 2019). Parallel mainstream schooling and supplementary tutoring will be the main leverage for policy decisions. We propose that if mainstream school class efficacy needs to be guaranteed, supplementary tutoring is needed to fill any deficiencies in individual academic performance in a rational way rather than *via* simplistic educational productization.

## Data Availability Statement

The original contributions presented in the study are included in the article/supplementary material, further inquiries can be directed to the corresponding author/s.

## Author Contributions

All authors listed have made a substantial, direct, and intellectual contribution to the work and approved it for publication.

## Funding

The National Social Science Fund of China 2020 Pedagogy general research topic “A study of educational discipline from a phenomenological perspective” (Topic Number BAA200027).

## Conflict of Interest

The authors declare that the research was conducted in the absence of any commercial or financial relationships that could be construed as a potential conflict of interest.

## Publisher's Note

All claims expressed in this article are solely those of the authors and do not necessarily represent those of their affiliated organizations, or those of the publisher, the editors and the reviewers. Any product that may be evaluated in this article, or claim that may be made by its manufacturer, is not guaranteed or endorsed by the publisher.
